# Environmental and anthropogenic factors affecting the increasing occurrence of shark-human interactions around a fast-developing Indian Ocean island

**DOI:** 10.1038/s41598-018-21553-0

**Published:** 2018-02-27

**Authors:** Erwann Lagabrielle, Agathe Allibert, Jeremy J. Kiszka, Nicolas Loiseau, James P. Kilfoil, Anne Lemahieu

**Affiliations:** 1 UMR 228 ESPACE-DEV, Université de La Réunion, IRD, Parc Technologique Universitaire, 2 rue Joseph Wetzell CS 41095, 97495 Sainte-Clotilde Cedex, France; 2 Institute for Coastal and Marine Research, Nelson Mandela Metropolitan University, Port Elizabeth, 6031 South Africa; 3 UMR PVBMT, Université de La Réunion, Pôle de Protection des Plantes, 7 Chemin de l’IRAT, 97410 Saint-Pierre, France; 4 Groupe de recherche en épidémiologie des zoonoses et santé publique (GREZOSP), Faculté de médecine vétérinaire, Université de Montréal, 3200 Rue Sicotte, Saint-Hyacinthe, QC J2S 2M2 Canada; 5 Department of Biological Sciences, Florida International University, 3000 NE 151 St., FL 33181 North Miami, USA; 6 UMR 9175 MARBEC, IRD-CNRS-IFREMER-UM, Université de Montpellier, 34095 Montpellier, France; 7 Department of Anthropology and department of Ichthyology and Fisheries Sciences, Rhodes University, PO Box 94 Grahamstown, South Africa

## Abstract

Understanding the environmental drivers of interactions between predators and humans is critical for public safety and management purposes. In the marine environment, this issue is exemplified by shark-human interactions. The annual shark bite incidence rate (SBIR) in La Réunion (Indian Ocean) is among the highest in the world (up to 1 event per 24,000 hours of surfing) and has experienced a 23-fold increase over the 2005–2016 period. Since 1988, 86% of shark bite events on surfers involved ocean-users off the leeward coast, where 96% of surfing activities took place. We modeled the SBIR as a function of environmental variables, including benthic substrate, sea temperature and period of day. The SBIR peaked in winter, during the afternoon and dramatically increased on coral substrate since the mid-2000s. Seasonal patterns of increasing SBIR followed similar fluctuations of large coastal shark occurrences (particularly the bull shark *Carcharhinus leucas*), consistent with the hypothesis that higher shark presence may result in an increasing likelihood of shark bite events. Potential contributing factors and adaptation of ocean-users to the increasing shark bite hazard are discussed. This interdisciplinary research contributes to a better understanding of shark-human interactions. The modeling method is relevant for wildlife hazard management in general.

## Introduction

Human–wildlife conflicts result from the negative impacts of interactions between wild animals and people. Mitigating human-wildlife conflicts is a growing challenge, particularly since the spatial and temporal overlap between wildlife and humans is increasing^1–3^. Understanding the environmental drivers of these interactions is critical for public safety and management^2,3^. In several cases, conflicts between different public interests can arise, and human-wildlife conflicts can adversely affect economic activities^1^. In the marine environment, this issue is exemplified by growing concerns surrounding shark bites on ocean-users^4^.

Over the past three decades, the number of unprovoked shark bites on humans has increased around the world, creating numerous challenges for coastal management policies^4^. This increasing trend has been documented in multiple countries^5^, and has been anecdotally linked to increasing recreational activities^6^, particularly surfing^5,7,8^. Despite receiving large amounts of media and public attention, shark bites remain relatively rare events^9^. Nevertheless, given urgent public demand for safety measures following these occurences^5^, multi-disciplinary research on shark-human interactions (SHI) is needed to develop effective shark risk mitigation policies^10^.

The systematic recording, description, and analysis of SHI has sparked scientific interest worldwide, including in Africa^11,12^, Australia^6^, North and South America^8,13,14^, French Polynesia^15^ and New Caledonia^16^. Forensic analyses have made it possible to often identify shark species involved in bites^17,18^. However, causative factors, including the potential influence of environmental variables on SHI, have only recently been investigated^8,19^. Preliminary research documented temporal (i.e., season, time of day)^18^ and physical variables (i.e., depth^6^, substrate type^20,21^ water temperature^22–25^, oceanic and atmospheric conditions)^6^ that may influence the likelihood of SHI.

Given that the probability of a shark bite event is directly influenced by the number of people entering the ocean^6,26^, it is necessary to first standardize bite incidences as rates (rather than absolute counts) before spatiotemporal trends can be examined^26,27^. Trends in shark bite incidence rate (SBIR) can be used to explore links between environmental variables and risks of human-shark interactions^8,17^. However, the number of studies using this approach remains rare, perhaps due to the lack of ocean-user and environmental monitoring data over the same spatial and temporal scales^17,26,27^.

Shark bites on humans have been documented throughout the history of La Réunion (France, Southwest Indian Ocean), but not anywhere near the levels experienced in recent years^28^. From 2010 to 2017, 23 shark bites occurred (see method section for inclusion criterion) around La Réunion, including 9 fatal events. This situation had a dramatic impact on ocean-based recreational activities, and has created what the media described as a “shark crisis” involving ocean-users, scientists, and management authorities^28,29^.

In this study, we investigated the relationships between environmental variables and human behaviours on the SBIR, particularly surfing, around the island of La Réunion from 1980 to 2016.

### Study site

**Figure 1:**
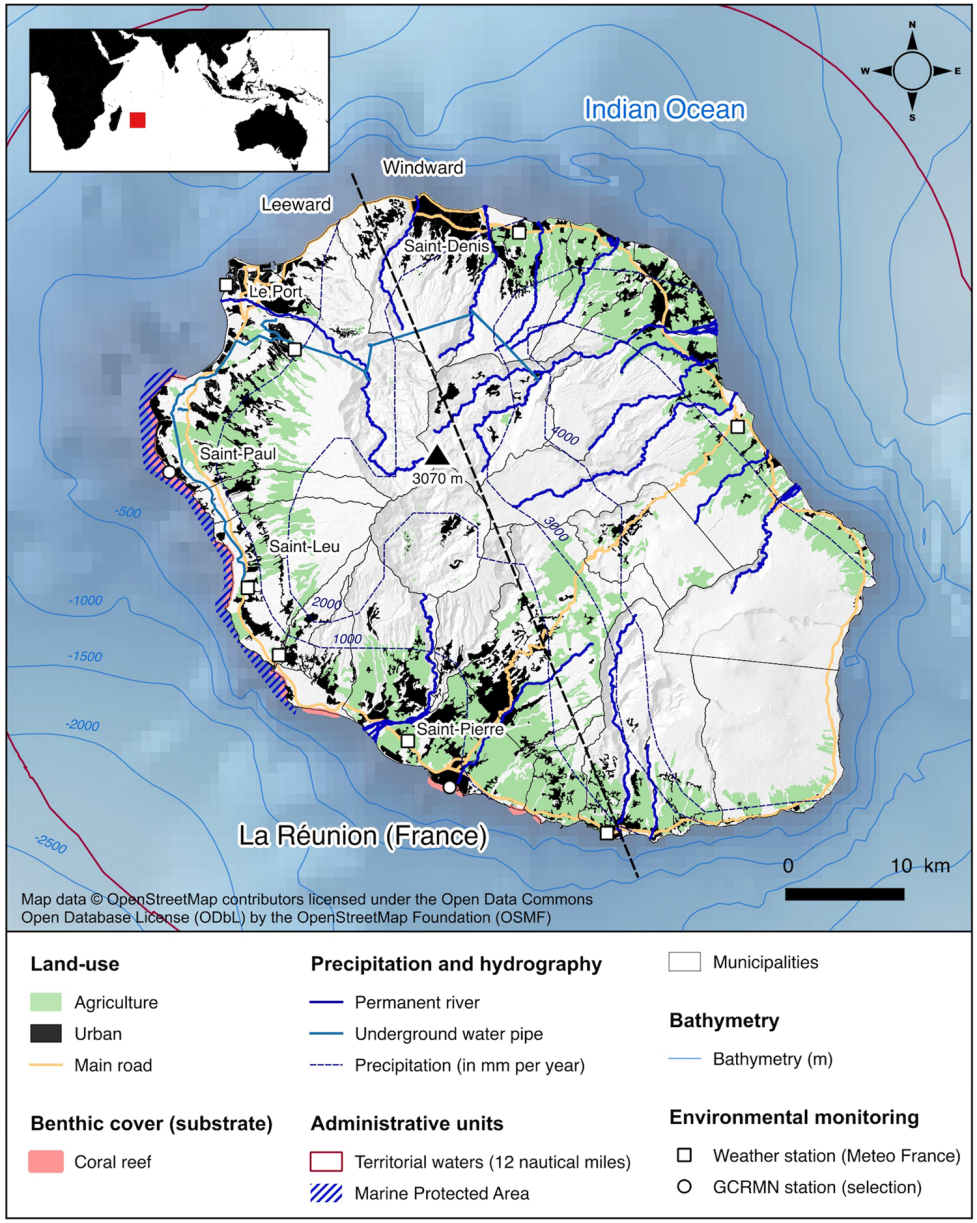
Map illustrating the location and spatial organization of La Réunion in the western Indian Ocean. The map was generated by EL. Altitude contour lines and shaded relief were generated using publicly available geographic data from the General Bathymetric Charts of the Oceans (The GEBCO_08 Grid, version 20100927, http://www.gebco.net). Data on land-use, benthic cover, hydrography, precipitation and administrative units were extracted from OpenStreetMap (http://www.openstreetmap.org). OpenStreetMap is open data, licensed under the Open Data Commons Open Database License (ODbL) by the OpenStreetMap Foundation (OSMF). GCRMN stands for Global Coral Reef Monitoring Network. The map was generated using QGIS 2.18 software (GNU licence).

La Réunion is a French volcanic island located in the tropical Southwest Indian Ocean (Fig. 1). The island is characterised by a narrow insular shelf (<5 km wide), and a steep topography that results in considerable run-off during the rainy season. The windward coast experiences high rates of precipitation annually (i.e., annual rainfall >3 m). Around the island, major marine habitats include coral reefs (~25 km long, Fig. 1), rocky coasts on the westward side (~100 km long), and soft bottom (sandy and muddy) coastal sediments that are mixed with small basaltic blocks (~90 km long)^30^. La Réunion is on the path of the westward South Equatorial Current, characterised by warm and nutrient-poor waters^31^. Over the last three decades, La Réunion has been characterised by rapid land-use changes and intensification of human activities, fuelled by demographic growth and economic development^32^. From 1980 to 2016, the human population in La Réunion increased from 500,000 to 850,000 (+67%) and urban areas expanded from 59 km^2^ to 260 km^2^ (+287%; estimated using updated GIS data and satellite images^32^; Supplementary Fig. S1).

Ocean-based activities in La Réunion include fishing and a wide range of water sports. Most fishing effort in the coastal waters of La Réunion comes from small fishing vessels (<12 m)^33^. Since 2007, 80% of fringing coral reefs located along the western and southern coast are protected within the *Réserve Naturelle Nationale Marine de La Réunion* (RNNMR, Fig. 1). Coastal tourism and leisure activities are concentrated on the leeward side of the island (west)^28^. Tourism grew rapidly from 120,000 tourists in the early 1980s to 400,000 since the early 2000s (+330%; La Réunion Tourism Board).

Surfers, bathers, and spear-fishers are among the ocean-user groups most exposed to the potential for SHIs^8^. Imported during the 1960s in La Réunion, surfing is practiced across approximately 50 distinct locations (grouped into 34 broader surfing sectors), including 40 within the boundaries of the RNNMR. The surfer population was estimated at 10,000 in the 2010s, while windsurfing and kitesurfing had only approximately 500 regular users during the same period^34^. Since 2014, 46 recreational scuba diving operators have been active in La Réunion (including 21 commercial), mostly on the west coast (87%), for a total of 140,000 annual dives. In 2002 the density of spear fishers on the island was estimated to be a maximum of 0.5 spear fishers per day per km of coastline along the leeward coast^35^.

## Results

### Shark bite records and incidence per ocean-user group (1980–2016)

**Figure 2:**
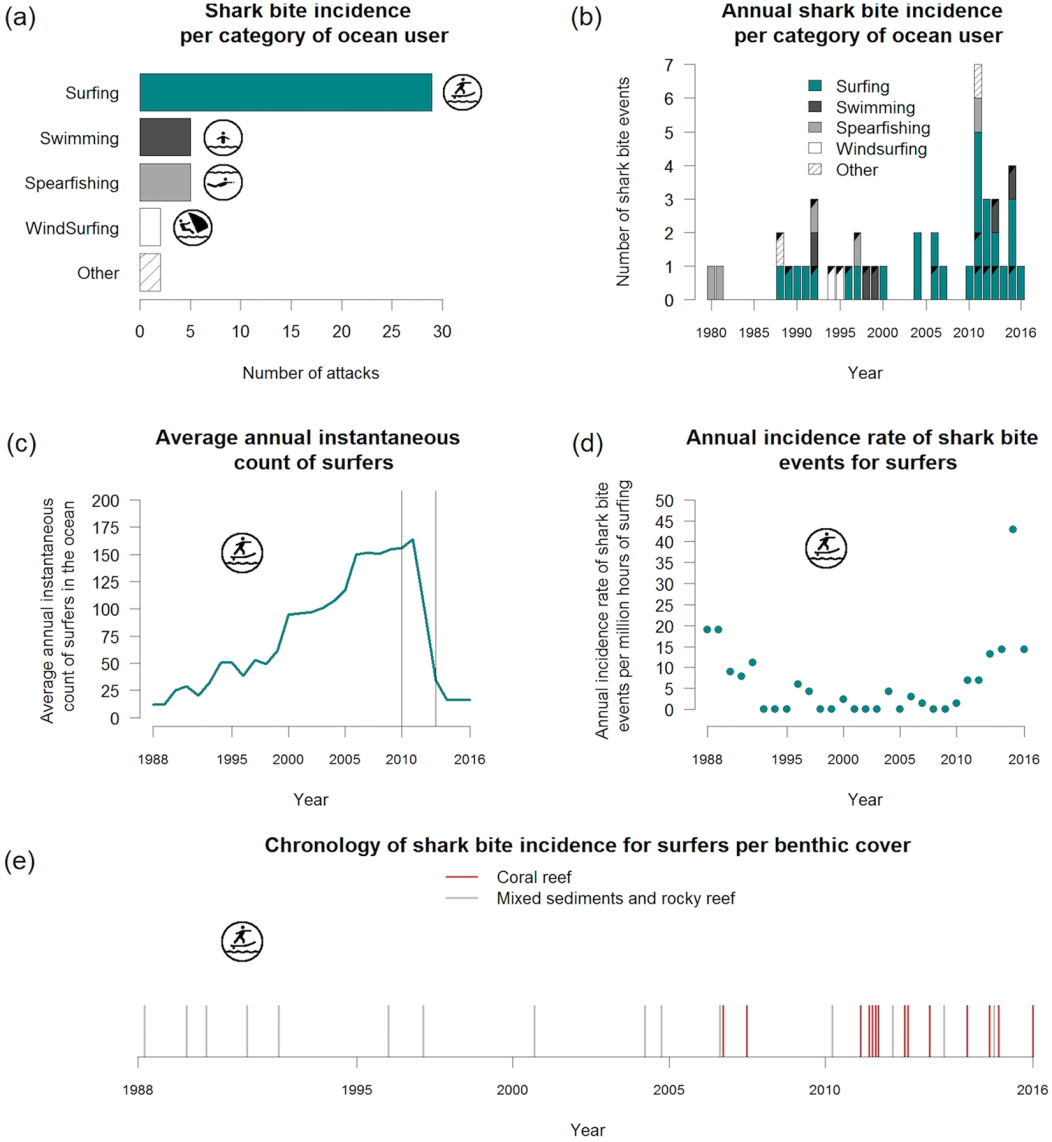
Shark bite incidence and incidence rate from 1988 to 2016 in La Réunion (43 events selected over 51 recorded). Incidence of shark bite events per category of coastal ocean-users (**a**) and per year (black triangles indicate fatal events) (**b**), reconstructed average annual instantaneous count of surfers (ICS) (the two vertical lines indicate the aerial survey period 2010–2013) (**c**), annual shark bite incidence rate for surfers (per million hours of surfing) (**d**) and the chronology of shark bite incidence for surfers (n = 29 events) per benthic substrate cover (coral reef in red) (**e**).

Of the 51 SHI recorded during the study period (1980–2016), 43 shark bite events fitted the criteria for inclusion in the study (Fig. 2). Of the 43 shark bite events, 67% involved surfers (n = 29), with the remaining 33% involving swimmers (n = 5), spear fishers (n = 5), windsurfers (n = 2), a gillnet fisher (n = 1), and a kayaker (n = 1; Fig. 2a). Across all activities, 42% of bites were fatal. However, this proportion reaches 64% for non-surfers (Fig. 2b). Shark bite events involving swimmers and windsurfers were all fatal. The case fatality rate (CFR) for isolated victims (i.e., no other person within 100 m) was 59% (n = 11) compared to 32% (n = 7) for non-isolated victims. The average age of victims across all activities was 29 ± 1.78 (mean ± SEM) and 28 ± 2.04 for surfers only. The sex-ratio (F:M) of victims was 2:12 for non-surfers, while all surfers were males. The species of shark involved was documented for 18 cases (42% of all events), including 12 events on surfers. Recorded species were *Carcharhinus leucas* for 11 cases (61%) and *Galeocerdo cuvier* for 5 cases (28%). The remaining two cases involved *Carcharhinus albimarginatus* and *Carcharhinus amblyrhynchos*. For the 12 shark bite events on surfers, 72% of cases involved *C*. *leucas*, with the remaining identified as *G*. *cuvier*.

Over the 1980–2016 period, the mean incidence of shark bites for all categories of ocean-users was 1.2 ± 0.2 events per year, with a positive annual rate of change of 0.05 (r^2^ = 0.14, p = 0.01), and a peak-value of seven events in 2011 (see Supplementary Fig. S2). The first shark bite on a surfer was recorded in 1988, and 52% of bites on surfers occurred between 2010 and 2016. The mean incidence of shark bites on surfers was 0.8 ± 0.2 event per year, with a positive annual rate of 0.06 (r^2^ = 0.26, p < 0.01) and a peak-value of five events in 2011.

### Abundance of surfers and shark bite incidence rate for surfers (1988–2016)

The total estimated surfing activity along the coastline of La Réunion over the 1988–2016 period is 9.5 millions hours. The average instantaneous count of surfers (ICS) over this period in La Réunion was 74.3 ± 9.7. The mean ICS per surf sector (n = 34) was 2.2 ± 0.7 with a maximum of 15.8 (in the surf sector of Saint-Paul - Trois-Bassins, on the west coast). From 1988 to 2011, the ICS increased 14-fold from an estimated minimum of 12 in 1988, to a maximum of 163.4 in 2011 (Fig. 2c). From 2011 to 2014, the mean ICS decreased 10-fold to 16, similar to its levels during the 1990s.

The vast majority (96.4%) of the ICS was distributed along the leeward coast, on coral reef-associated substrate (concentrating 75.1% of the total ICS) and mixed sediments/rocky reef (21.3% of the total ICS). Surfing on the windward coast (3.6% of ICS) is marginal. There were no marked seasonal (summer: 52%, winter: 48%) nor diel (morning: 51%, afternoon: 49%) patterns of ICS detected over the 2010–2012 period (aircraft-based survey results). However, since 2012, surfers have been observed practicing earlier in the morning (57% of daily ICS occurred in the morning in 2012, compared to 45% in 2010).

**Figure 3:**
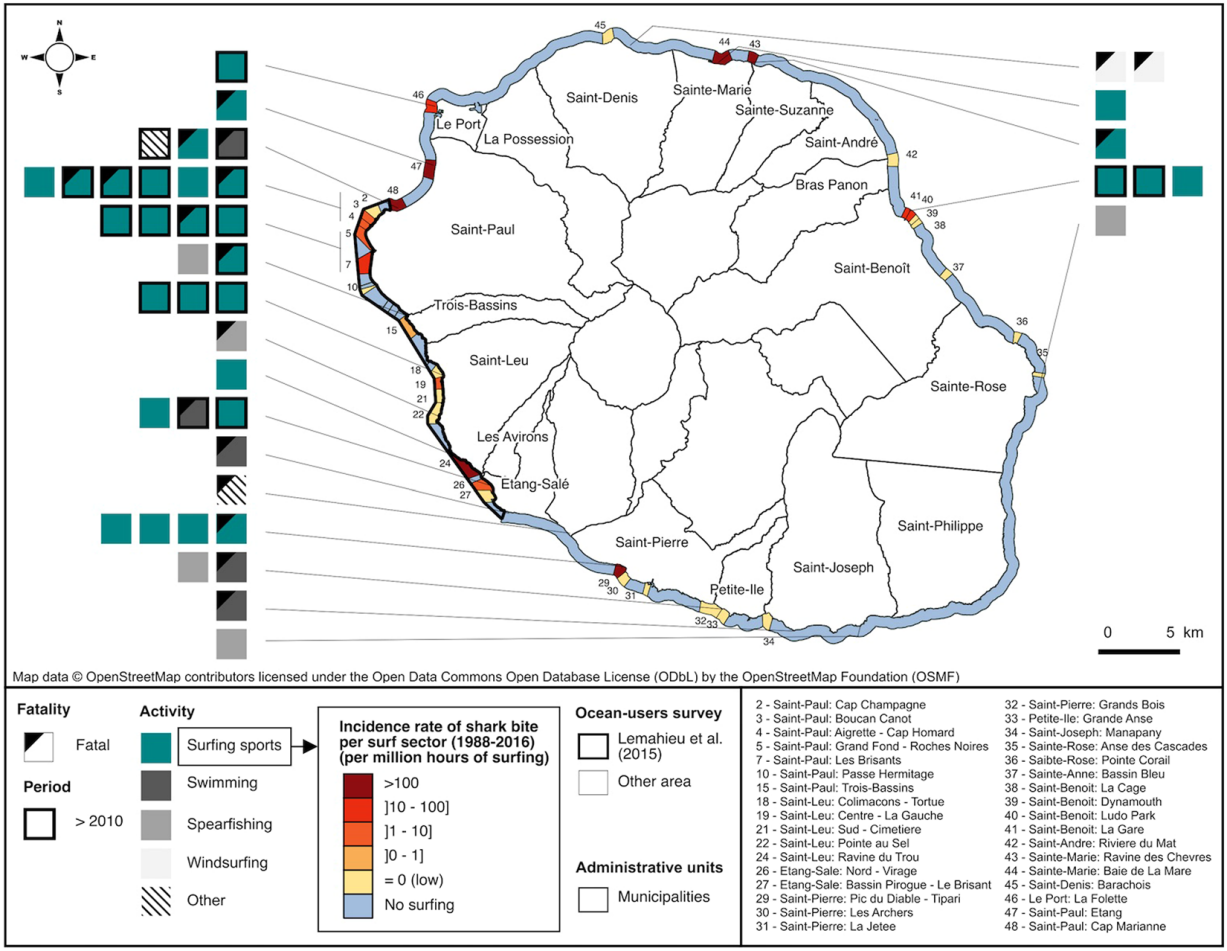
Spatial distribution of the 43 studied shark bites events (1988–2016) in La Réunion. Shark bite rates for surfers over the 1988–2016 period are provided per sector. Data on administrative units were extracted from OpenStreetMap (http://www.openstreetmap.org). OpenStreetMap is open data, licensed under the Open Data Commons Open Database License (ODbL) by the OpenStreetMap Foundation (OSMF). The map was generated using QGIS 2.18 software (GNU licence).

The mean annual SBIR for surfers from 1988 to 2016 was 6.5 ± 1.7 per million hours of surfing (pmh; Fig. 2d). The SBIR decreased from 19 pmh in 1988 to 0 pmh in 1993. During the 1993–2010 period, the mean annual SBIR remained relatively low (1.3 ± 0.5 pmh), but it increased from 2011 to reach a maximum of 42.8 pmh in 2015 (1 event per 24,000 hours). Over the 1988–2016 period, the SBIR per surfing sector (i.e., surf spot or group of surf spots) ranged from 0 (no event) to more than 1,000 pmh. Highest SBIR per surfing sector occurred where a single shark bite event was associated with a very low ICS (Fig. 3).

### Environmental conditions associated with shark bites on surfers (1988–2016)

**Figure 4:**
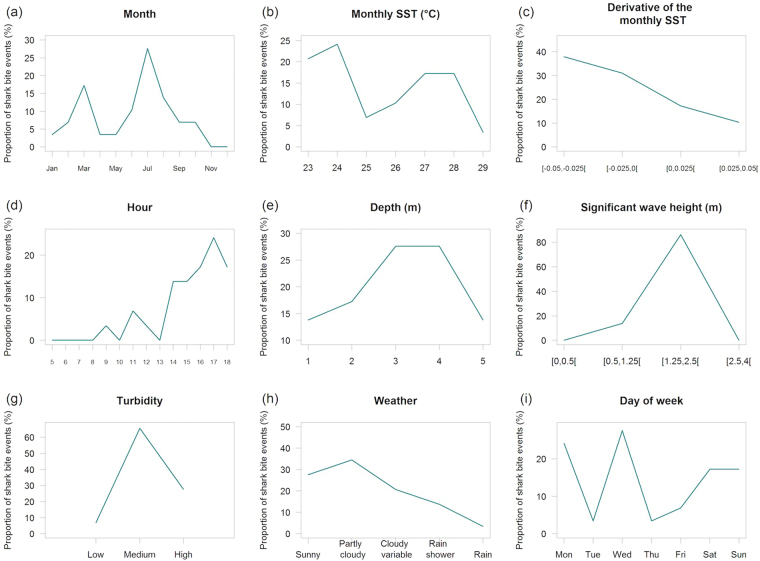
Frequency distribution of shark bites incidence (surfers only) across nine temporal and environmental variables: month (**a**), monthly SST (**b**), derivative of the monthly SST (**c**), hour (**d**), depth (**e**), significant wave height (**f**), turbidity (**g**), weather (**h**) and day of week (**i**).

Overall, 86% of bites occurred on the leeward coast, against 14% on the windward coast (Fig. 3). Of the 16 events that occurred during the 2010–2016 period, 90% (14) were distributed along a 50 km stretch of the leeward coast, between Le Port and Etang-Salé (Fig. 3). Shark bites have occurred year round, with the exception of November and December (Fig. 4a). Nearly half (48%) of bites occurred from June to August, with a peak in July (28%). The mean sea surface temperature (SST) at bite sites was 25.5 ± 0.28 °C with a range of 23.0–28.7 °C (median = 25.0 °C; Fig. 4b). The majority of bites were associated with negative monthly SST derivative values (i.e., cooling water; Fig. 4c). Most bites occurred in the evening (76% after 16:00; Fig. 4d), and at depths between one to five meters (Fig. 4e) in the surf zone. A large portion of bites (48%) occurred in coral reef habitats (Fig. 2e).

Sea state was moderate to rough (1.25 m to 4 m significant wave height SWH) in 97% of cases (Fig. 4f). Of the 26 cases with an estimated turbidity value, 7.7% occurred in low turbidity, 65.4% occurred in medium, and 26.9% occurred in high turbidity water (Fig. 4g). The weather was “Sunny” to “Cloudy variable” during 83% of the 29 shark bite events on surfers (Fig. 4h). In addition, most events occurred on Mondays, Wednesdays and during the week-end (Fig. 4i).

### Modelled shark bite incidence rate for surfers (1988–2016)

**Figure 5:**
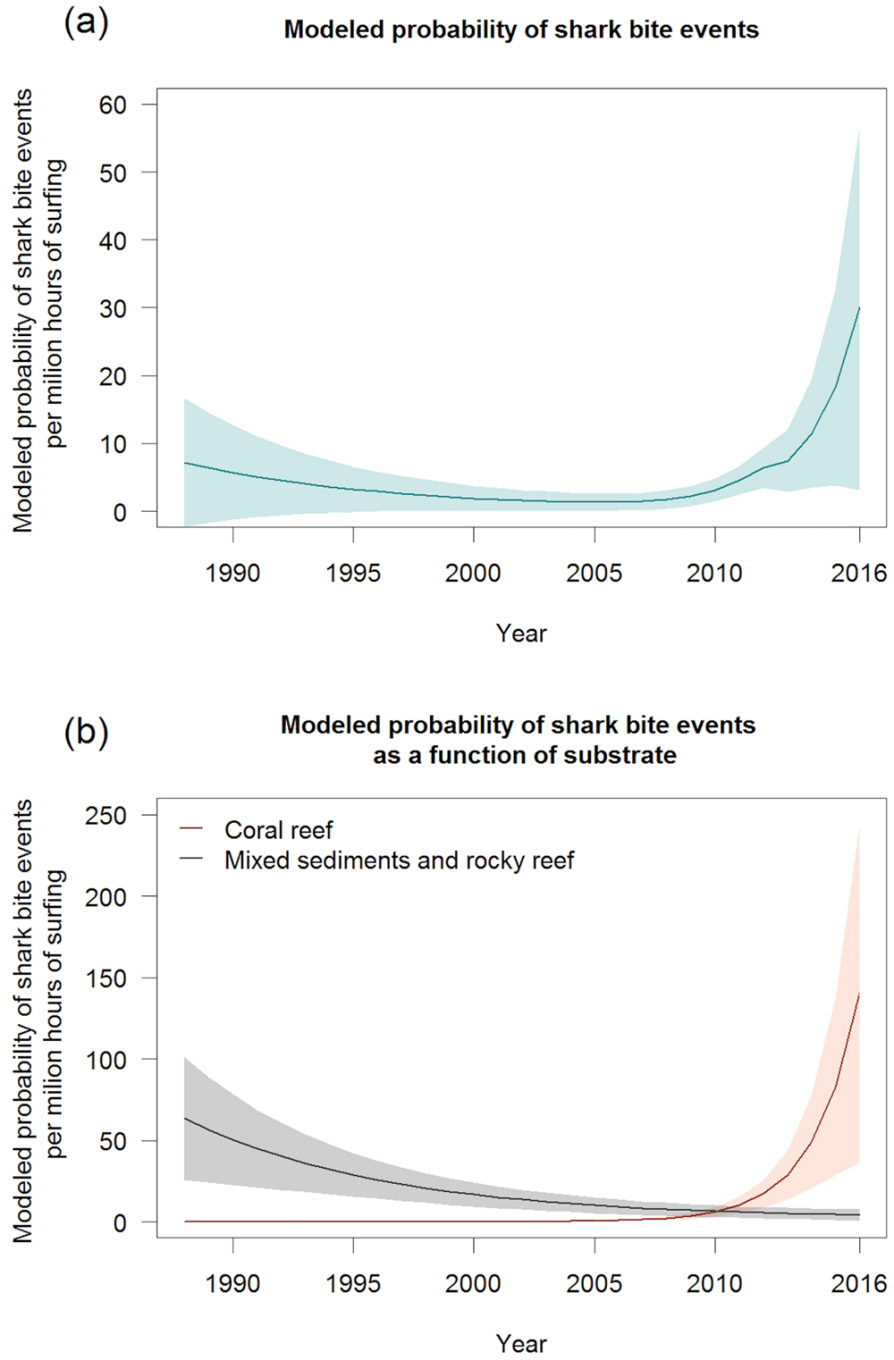
GLM-based prediction of shark bite incidence rate for surfers (per million hours of surfing) as a function of years (**a**) and substrate type (**b**) over the 1988–2016 period.

Assuming a uniform distribution of ICS across space, time and environmental conditions, Generalized Linear Model (GLM) results (Table 1) showed that modelled SBIR decreased 8-fold from 31.7 ± 18.9 pmh in 1988 to 4.9 ± 2.6 pmh 2007, and then increased 15-fold to 72.5 ± 53.8 pmh in 2016. The mSBIR corrected by the proportion of ICS over substrate types (coral or other) in La Réunion decreased 5-fold from 7.1 ± 9.5 pmh in 1988 to 1.3 ± 1.3 pmh in 2005 (Fig. 5a). Then, it increased 23-fold to 30.0 ± 26.9 pmh in 2016.

**Table 1:** Analysis of deviance table for the generalized linear model of shark bite incidence rate in La Réunion.

	Estimate	Df	Deviance	Residual Df	Residual Dev	P-value	
(intercept)	−964.4 ± 213.9			1391	259.8	—	—
Year	0.5 ± 0.1	1	0.3	1390	259.4	0.560	
Substrate	1187.7 ± 221.9	1	8.3	1389	251.2	0.004	**
Period of day	−283.0 ± 224.8	1	7.0	1388	244.1	0.008	**
Derivative of the monthly SST	−17.3 ± 5.3	1	8.3	1387	235.8	0.004	**
Substrate:Year	−0.6 ± 0.1	1	44.2	1386	191.6	2.9E-11	***
Year: Period of day	0.1 ± 0.1	1	2.4	1385	189.2	0.120	

Df: degree of freedom, Dev: deviance. P-value significance codes: 0 ‘***’ 0.001 ‘**’ 0.01 ‘*’ 0.05 ‘.’ 0.1 ‘’ 1.

**Figure 6:**
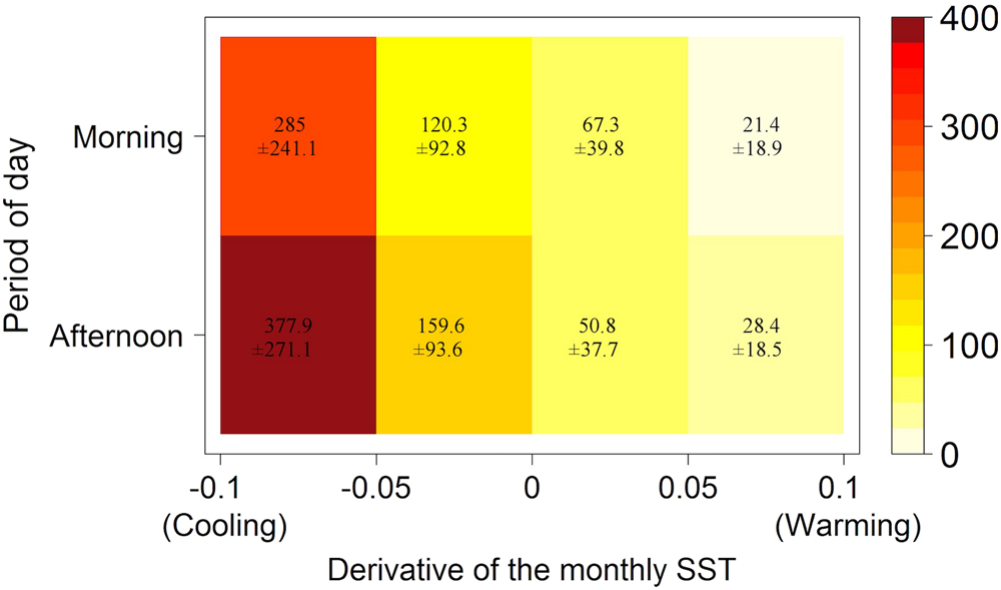
GLM-based prediction table of shark bite incidence rate for surfers (per million hours of surfing) for the year 2016 on coral reef substrate as a combined function of day period (morning-afternoon) and of the derivative of the monthly SST (cooling-warming).

The mSBIR was positively correlated with substrate type (estimate = 1187.7 ± 221.9). The interaction between year and substrate type was positively correlated, indicating a shift in shark bite events from mixed substrate habitats, toward coral reef habitats since the early 2010s (estimate = −0.6 ± 0.1; Fig. 5b). The mSBIR decreased 15-fold on mixed sediments and rocky reef from 63.4 ± 37.9 pmh in 1988 to 4.3 ± 3.5 pmh in 2016. On coral reef habitats, the mSBIR rose from 1.4E10–4 ± 3.5E10–4 pmh in 1988 to 140.8 ± 104.0 pmh in 2016. The mSBIR was also influenced by the period of day (estimate = −283.0 ± 224.8; Fig. 6). In 2016, the average mSBIR was 1.3-times lower in the morning (62.3 ± 38.3 pmh) than in the afternoon (82.7 ± 41.1 pmh). Shark bite events were negatively correlated to the derivative SST (estimate = −17.3 ± 5.3 pmh). For instance, for the year 2016 on coral reefs, the average mSBIR was 14-times higher during month with a derivative SST value < −0.05 (338.0 ± 195.3 pmh) than during month with a derivative SST value > 0.05 (25.7 ± 14.1 pmh). The month, monthly SST, cumulative rainfall periods and moon phase were not significantly related to the mSBIR. The area under the curve (AUC) of the receiver operating characteristic (ROC; 0.81) indicates a strong predictive power of the model.

## Discussion

Over the 1980–2016 period, La Réunion was in the top-6 list of global hotspots for unprovoked shark bites reported in a single administrative unit (state or territory^29^). The CFR of 42% for all users and 31% for surfers is very high compared to other countries, such as Australia (CFR = 13% over the 1980–2009 period^6^). Similar to Florida, California, Brazil, South Africa, and Australia^29^, surfers are the users that are most exposed to shark bite incidents in La Réunion. The CFR was two-times higher for isolated surfers, confirming recent clinical study findings in La Réunion^36^.

The increasing number of ocean-users (mostly surfers) is likely an important driver of the increasing shark bite incidence in La Réunion during the 1988–2016 period^29^. Indeed, higher shark bite incidences occur during non-school and work days (Monday, Wednesday and week-end), when ocean users abundance is higher^28^. This result is compatible with the hypothesis that a higher abundance of ocean-users increases the probability of a shark bite^19^. During a first phase (1988–2005), the mSBIR for surfers (corrected by the spatial distribution of the mean annual ICS across surf spots) decreased 5-fold. One possible explanation for this downward trend could be surfers learning to avoid, through “trial and error”, areas with high concentrations of potentially dangerous sharks (PDSs^19^) off the windward coast. Similar findings have been documented in California, where declining shark bite rates were attributed to increased knowledge by ocean-users on how to minimize risk through space and time selection criterion^8^. In a second phase (2005–2016), the mSBIR for surfers increased 23-fold, peaking to 30 ± 26.9 shark bite events per million hours of surfing. As a benchmark for comparison, in California, the mSBIR was of 0.005 per million hours of surfing in 2013^8^ (assuming 12 hours of surfing uniformly distributed over each day period). The mSBIR increase in La Réunion during the early 2010s seems so rapid that it exceeded the adaptation rate of ocean-users faced with an unprecedented high level of shark bite risk, resulting with a peak of 5 shark bite events in 2011.

The spatial shift in shark bite events from mixed substrate habitats toward coral reef habitats is most likely explained by the desertion of the windward coast by surfers since the mid-1990s and their subsequent aggregation on coral surf spots off the leeward coast, combined with an increasing abundance/presence of PDSs on this area during the early 2010s. Shark bites have occurred year-round in La Réunion, with the exception of November and December, but were more frequent during winter (i.e., from March to August). This seasonal pattern is consistent with the results of a recent study on the movements of bull sharks in the leeward coastal waters of La Réunion^37^. Together, these findings suggest that a high risk of shark bite is correlated with a higher occurrence of bull sharks during the austral winter in this area. However, no data are currently available on the occurrence and habitat use of bull and tiger sharks along the windward east coast, as studies have focused solely along the leeward coasts to date^37^. Off Mozambique and on the east coast of South Africa, bull sharks are known to undertake seasonal migrations from tropical and subtropical waters to temperate latitudes during summer^23,38^. Movements of up to 2,000 km between the Seychelles and Madagascar suggest the existence of transoceanic movements of bull sharks between Indian Ocean islands, including La Réunion^39^. Moreover, diel patterns of shark bites in La Réunion are compatible with the hypothesis that bull sharks move closer to shore for foraging when the sunlight decreases^40,41^.

Factors underlying the increasing SBIR include an increasing abundance of large coastal sharks (particularly bull sharks)^29^. Potential contributing factors to this increasing shark abundance encompass biophysical changes that alter marine and coastal habitats, water quality, and the distribution/abundance of prey species^29^, including species targeted by commercial fisheries. Coastal ecosystems have been significantly altered by human activities over the last decades in La Réunion^42–44^, similarly to Hawaii^45^. Coastal and deep demersal fish stocks are considered overexploited since the early 2000s in La Réunion^33^ and this could have decreased shark prey species abundance (see Supplementary Fig. S1). It is also possible that ecosystem changes along the west coast of La Réunion may have created more suitable habitat conditions for bull sharks (as has been seen in other parts of the world^21,46–49^), enhancing their occurrence and the risk of interaction with humans in near shore waters. Ports and raw sewage channels constructed on the leeward coast over the last 40 years in La Réunion provide potential suitable habitats for bull sharks, including juveniles, for which the occurrence in the shallow waters and in rivers has been increasingly observed in recent years^37^. The east to west transfer of 15 million cubic meters of freshwater per year since 1999 may also have contributed to increased freshwater outflow that modified the physicochemical properties of coastal water masses. The east-west water transfer project was implemented to support agriculture development in the drier leeward coast. However, a third of the water transferred from the windward coast is currently distributed for domestic use and is discharged off the leeward coast. Although urban sewage water is increasingly processed in treatment plants that were built in the 2000s, it has also created artificial point-source freshwater outflow along this coast. It was demonstrated that higher groundwater discharge caused lower salinity (34.8 PSS) on a fringing coral reef section along the leeward coast of La Réunion in Saint-Paul^50^. In the same area, long-term exposure to elevated nitrate or ammonium levels (5–20 μmol l^−1^) was observed in nutrient-enriched groundwater discharge with terrigenous inputs^51^. Likewise the Global Coral Reef Monitoring Network (GCRMN) method applied in La Réunion showed an increase in algal cover on coral reef along the leeward coast since the early 2000s^43^ (see Supplementary Fig. S3). Unfortunately, in the absence of long-term data on coastal shark abundance in La Réunion, it is currently impossible to attribute higher shark bite incidences to a single causative factor. However, a combination of local factors that affected PDSs densities is most likely suspected^28,37^.

Since 2010, the increasing occurrence of shark bites led to the implementation of a number of risk mitigation measures. A total ban on swimming and surfing was introduced in 2013^29^. Shark nets were implemented at two beaches^28^. A shark-patrol system involving immersed shark spotters has been operational since 2015 (“*Vigie Requin Renforcée*”). In 2016, the Reunion Shark Risk Management Centre (CRA) was created to coordinate public authorities, stakeholders, ocean-users and experts in shaping future shark-risk mitigation measures in La Réunion. The so-called “shark crisis” in La Réunion polarised antagonistic opinions and social conflicts^10,52^. Fueled by controversies publicised through the press and social networks, conflicts have arisen about the lack of, or slow progresses towards, shark-risk management strategies being implemented in La Reunion. Heavy debates focus on shark fishing activities as a protective measure^14^, including the deployment of drumlines inside a MPA (*Réserve Naturelle Marine de La Réunion*).

In a situation where decision makers tend to manage public emotions^53–55^ rather than the hazard itself^10^, efficient management of negative human-wildlife interactions need to be based upon scientific evidences^54^. To that purpose, our analytical framework can be used to simulate wildlife-related hazard management scenarios (e.g., spatial-temporal changes of human uses), to set quantitative baselines for management policies and to determine a realistic and socially acceptable level of risk. Overall, this interdisciplinary research highlights the importance of social-ecological monitoring and contributes to better understand shark-human interactions. The method is also relevant for wildlife hazard management in general.

## Methods

### Conceptual framework

This study uses the classification of SHI of Neff and Hueter (2013)^56^. A shark bite is an incident where a shark bites a human (or an inanimate object holding or held by that person) resulting in minor, to severe injuries. A fatal SHI is an incident resulting in death. A shark encounter is a close (<1 meter) or direct contact without bite between a shark and a human. We chose to exclude shark encounters from the scope of this study, instead focusing only on shark bite events due to the high reporting biases of the former.

To examine the potential influence of environmental conditions on the occurrence of SHIs, we adopted an existing statistical framework^8^ (see Supplementary Fig. S4). A stable reporting effort of shark bite events is assumed. The probability of a shark bite on human (P), within a given area and time frame depends on the abundance of sharks (S), the abundance of ocean-users (U), and the overlap (O) between oceans users and sharks. We also hypothesize that (S), (U) and (O) are influenced by the environmental factors (E). Since shark bites are discrete rare events, the number of bite events per unit of space and time can be a function of covariates reflecting S, U, O and E^8,57^. We used this probability framework to analyze shark bite events recorded between 1980 and 2016 in La Réunion, coupled with information on ocean-use (particularly surfing) and the state of the environment (see Supplementary Fig. S5). We chose to focus our analysis on surfing because this activity is the most frequently exposed to shark bites, and quantitative monitoring data were available^28^.

### Selection, description and mapping of shark bite records

The oldest documented case of shark bite on human in La Réunion was reported in 1904 (see Supplementary Fig. S6) with rare and sporadic incidences reported by local authorities and in newspapers through 1980. Since 1980, local archives suggest that newspapers have consistently documented shark-human interactions (particularly bites). Accordingly, shark bite occurrence data were extracted from the Global Shark Attack File (GSAF) on-line database, as well as from local scientific reports or publications^36,58–60^ for subsequent analyses. Additionally, these data were supplemented by conducting a systematic search of records in newspaper articles during the entire study period (1980–2016). Each shark bite report was documented using a compilation of scanned local paper press releases (published before 2010) and several informal interviews with local ocean-users (divers, surfers, spear fishers) completed with a continuous on-line local press survey undertaken from 2011 to 2016. Modifications of the original GSAF dataset were also undertaken, including the addition of missing events, the removal of double counted events, and the correction of dates and locations. The following minimum set of basic descriptors were documented for each reported shark bite event: time-date location, injuries (including fatal injuries), victim age, gender, and activity. Data were anonymized and a unique ID number was attributed to each event. The CFR^61^ was calculated as the proportion of fatal cases. The activities practiced by the victims were categorized as follows: (1) surfing (and other surfing sports, except windsurfing), (2) spearfishing, (3) bathing/swimming/wading (including after a fall at sea), (4) windsurfing, (5) diving, (6) fishing and (6) kayaking. The presence of other ocean-users in the water within a radius of approximately 100 m during the shark bite event was also documented. Where shark species information is mentioned (victim and/or witness account), only cases providing a positive species identification are assessed. Mapping of shark bite events was based on interviews with victims and witnesses, and the local knowledge of authors of the present study (EL, NL). Google Earth aerial photography and a 5 m resolution bathymetric model Litto3D®-HYDRORUN (provided by Institut Français de Recherche pour l’Exploitation de la Mer, IFREMER) were used as background information for mapping in QGIS^62^ Version 2.18 software.

### Surfer abundance survey

Since most shark bite events were recorded on surfers in La Réunion, we chose to focus this study on this segment of the population of ocean-users. Mean instantaneous count of surfers (ICS) were extracted from an aircraft-based ocean-users survey covering the 2010–13 period^28^. The initial purpose of this aerial photography survey was to provide an estimate of ocean-user abundance to guide the RNNMR management. During this period, 164 aerial surveys were carried out to count ocean-users, including surfers, along the 40 km stretch of coast encompassing the RNNMR between Etang-Salé in the south to Boucan-Canot in the north^30^ (Fig. 1). This stretch of coast was divided into 27 sectors, including 14 sectors with one or more surf spots. Those 14 sectors represent a major proportion (estimated to 81%) of the surfing effort in La Réunion^28^. Ocean-users were counted using aerial photographs taken from planes during 50 minute roundtrips flights at an altitude of 300 m over the RNNMR. Flights were evenly distributed over time (holidays/work, week/week-end) to assess and compare the densities of ocean-users during summer (November-April) and winter (May-October), and throughout morning (at 10:30) as well as afternoon (at 15:30). The Supplementary Figs S7 and S8 provide an overview of the surveys methods and results^28^.

The 20 surfing sectors located outside of the aircraft-based survey area^28^ are distributed along a 180 km stretch of coastline. Out of Saint-Pierre (15% of the ICS), the others surf sectors contribute to 4% of the total ICS. Those areas are rarely surfed due to a higher perceived risk of shark attacks, and a lower frequency of good conditions for surfing. The ICS in those sectors were estimated based on interviews with local experienced surfers over the course of the last five years along the coastline of La Réunion. We asked them to provide their best estimate of the average number of days when surfing was possible (optimal sea conditions) *D* and the average number of surfers who would come to surf during these days *S* per surf spot for the year 2010 (i.e., before increased annual shark-human interactions). We then multiplied *D* by *S* by two hours (average estimated duration of a surf session) and divided this result by the number of hours when surfing conditions were option in a year (12 hours, 365 days) to obtain the estimated ICS per sector. The choice to distribute the abundance of surfers uniformly over 12 hours is based on the observed presence of surfers from sunrise to sunset, and low measured variation of surfer abundance between the morning and the afternoon (see Supplementary Fig. S8). The ICS in these 20 surfing sectors was extrapolated to the period 2010–2013 based on the mean abundance trend measured using aerial photography over the same period.

During the 1988–2009 period, the ICS per sector was inferred using trends of the number of surfers licensed at the French Surf League through the Surf League of La Réunion (see Supplementary Fig. S9). The number of licensed surfers in La Réunion increased 14-fold during this period. The use of this proxy is corroborated by the observation that from 2010 to 2013, the number of surf licensees followed a decreasing trend (−72%), similar to the ICS trend detected throughout the aircraft-based survey (−75%). For the 2014–2016 period, we assumed a stable ICS. Indeed, despite the ban on surfing proclaimed in 2013 (but only enforced from 2015), a residual population of surfers continues to be active.

A final quality control procedure of annual ICS data (all sectors combined) was conducted using the number of surfboards sold per year over the 1988–2016 period as an independent validation data set (see Supplementary Fig. S10). The correlation coefficient between the annual number of surfboard sales and the ICS is 90.6%.

### Shark bite incidence rate

Based on shark bite records, the mean annual shark bite incidence was calculated for the 1980–2016 period. The SBIR is a measure of the frequency at which shark bite events occur in a population exposed to the shark bite hazard. The counts of shark bites on surfers were transformed into an annual incidence rate by dividing the annual count of shark bites by the cumulated hours of surfing per year. The cumulated hours of surfing per year was calculated by multiplying the ICS by the number of daytime hours (12) and the number of days per year (365). The SBIR is expressed in number of shark bites per one million hours of surfing (SBIR = N_bite_/10^6^_h surf_).

### Environmental variables

A suite of nine environmental variables were selected to describe the environmental conditions given the presence of shark bite events on surfers: 1) depth^6^, 2) substrate type^21^, 3) monthly sea surface temperature (SST)^6,22–24^, 4) derivative of the monthly SST (to account for seasonal SST trends, i.e., cooling or warming), 5) moon cycles^24,25^, 6) cumulative rainfall^6^, 7) atmospheric conditions (cloud cover)^6^, 8) water turbidity^22^ and 9) wave height^6^. In a second step, to compare “incident” versus “no incident” conditions, continuous SST and rainfall time series covering the 1988–2016 period were used. However, continuous time series were not available for other variables (i.e., atmospheric conditions, cloud cover, water turbidity and wave height).

The substrate type at the shark bite event sites was derived from a benthic sediment map CARTOMAR® (BRGM, 2008). Two categories of substrate were distinguished: (1) coral reef (including coral patches on rocky reef) and (2) mixed bottoms (pebble, sand, mud, clay) and rocky reef.

Monthly mean SST data was provided by the NOAA/OAR/ESRL PSD, Boulder, Colorado, USA, from their Web site at http://www.esrl.noaa.gov/psd/, in area 20°S–22°S, 55°E–57E for the period (1988–2016)^63^. The derivative of the monthly SST was calculated to account for seasonal SST variations. The Supplementary Fig. S3 provides additional yearly ocean-scale SST-based indexes: the Dipole Mode Index (DMI)^64,65^ and the Central Equatorial Indian Ocean Index (CEI)^66^.

The lunar cycle was grouped according to the eight standard moon phases^24^: (1) full moon (2), waxing gibbous (3) first quarter (4) waxing crescent (5) new moon, (6) waning crescent (7) last quarter and (8) waning gibbous.

Daily rainfall data (in mm per day) were extracted from the online Meteo-France database using the closest weather station with a complete data series over the 1988–2016 period (seven meteorological stations distributed along the coastline, within a 10 km distance range from surf sectors). A set of cumulative rainfall period from 1 to 30 days before the day of shark bite event were calculated together with the corresponding mean over the same period per year from 1988 to 2016. The Supplementary Fig. S1 provides an overview of the major intense rainfall events that occurred during the 1980–2016 period.

Atmospheric conditions of the day were extracted from the online Meteo-France (French National Meteorological Service) database and assigned to one of the following broad categories: (1) Sunny, (2) Partly cloudy, (3) Cloudy variable, (4) Rain shower and (5) Rain - heavy rain. Significant Wave Heights (SWH) in the day of the incidents were extracted from daily marine reports (Meteo-France). Significant Wave Heights were reported into five broad categories using the Douglas sea scale: (1) smooth [0 m–0.5 m], (2) slight [0.5 m–1.25 m], (3) moderate [1.25 m–2.5 m], (4) rough [2.5 m–4 m] and (5) very rough [>4 m].

In the absence of long-term monitoring data, turbidity was assessed for each site using interviews with direct observers or information reported in newspaper articles. Turbidity is caused by suspended matter resulting from physical (e.g. erosion, tidal movements, waves) and biological processes (e.g. growth of phytoplankton)^22,67^. Water turbidity was classified in three categories: (1) low with a visibility of more than 10 m (use of terms “transparent”, “clear», “crystal clear”), (2) medium with a visibility comprised between 5 m and 10 m (“fairly clear”, “not very turbid”) and (3) high with a visibility inferior to 5 m (<<dark”, “brown”, “muddy>>, “dirty”, “chocolate”). Turbidity assessment data were validated using a Linear Discriminant Analysis (LDA; three classes) on three variables that may influence local turbidity^66^: wave, rainfall (five preceding days, chosen after initial testing of a set of time interval) and distance to the closest permanent river. The proportion of observed turbidity that were well predicted by the LDA was 60% for low turbidity, 75% for medium turbidity, and 60% for high turbidity. Although the turbidity assessment is only partially validated using the LDA method, we provide this data given the importance of this potential contributing factor on *C*. *leucas* presence and behavior.

### Data analysis

Linear regression was fitted to the shark bite incidence (for all categories of ocean-users and for surfers) to determine the rate of change during time (1980–2016), and to compare trends with other global shark bite hotspots^29^ (see Supplementary Fig. S2). In a second step, a linear regression was fitted to the SBIR on surfers (1988–2016). The environmental conditions of shark bite on surfers were described.

In a third step, a Generalized Linear Model (GLM^68^) assuming a binomial distribution with a logit-link function, was fitted to examine the relationships between shark bite events on surfers (1) and no bite events (0) and predictor variables (i.e., environmental variable) from 1988 (year of first bite event on surfer) to 2016. The model was built to investigate the potential effects of environmental predictors within the usual conditions of surfing (daytime, wave height inferior to four meters and depth inferior to five meters).

The GLM uses data spanning over a spatiotemporal array of 34 surfing sectors and 28 years, at the temporal resolutions of year, month and day period (morning-afternoon). The model was populated with (1) using shark bite incidence records and with (0) when no event occurred. The abundance of surfers was based on mean ICS estimates per surf sector per year. Monthly and half-day ICS estimates were proportionated using seasonal (winter:summer) and diel (morning:afternoon) ICS ratio deduced from the survey of ocean-users^28^. The model integrates environmental variables with a complete temporal coverage over the period (1988–2016): “depth”, “substrate”, “monthly SST”, “derivative of the monthly SST”, “cumulative rainfall periods” and “moon”. Temporal patterns (inter-annual, seasonal and diel) were investigated using the variable “year” (continuous variable), “month” and “day period”. We tested all the interactions between predictor variables. A stepwise model selection by Akaike Information Criterion (AIC) was then performed to choose the best model based on the lowest AIC. The significance threshold was set at p = 0.05. A Receiver Operating Characteristic curve (ROC curve) was built using predicted versus observed shark bite events. The predictive power of the model was then measured by the area under the ROC curve (AUC)^69^. Predictions are expressed as a probability of shark bite per million hours of surfing (pmh).

Analyses and figures were made using R^70^ software within the MASS^71^, pROC^72^ and Lattice^73^ packages. Maps were generated using QGIS^62^ Version 2.18 software.

### Data availability

The datasets generated and analysed during the current study are available from the corresponding author on reasonable request.

## Electronic supplementary material

Supplementary Information
