# Author Correction: Environmental and anthropogenic factors affecting the increasing occurrence of shark-human interactions around a fast-developing Indian Ocean island

**DOI:** 10.1038/s41598-018-28406-w

**Published:** 2018-07-04

**Authors:** Erwann Lagabrielle, Agathe Allibert, Jeremy J. Kiszka, Nicolas Loiseau, James P. Kilfoil, Anne Lemahieu

**Affiliations:** 1 UMR 228 ESPACE-DEV, Université de La Réunion, IRD, Parc Technologique Universitaire, 2 rue Joseph Wetzell CS 41095, 97495 Sainte-Clotilde Cedex, France; 2 Institute for Coastal and Marine Research, Nelson Mandela Metropolitan University, Port Elizabeth, 6031 South Africa; 3 UMR PVBMT, Université de La Réunion, Pôle de Protection des Plantes, 7 Chemin de l’IRAT, 97410 Saint-Pierre, France; 4 Groupe de recherche en épidémiologie des zoonoses et santé publique (GREZOSP), Faculté de médecine vétérinaire, Université de Montréal, 3200 Rue Sicotte, Saint-Hyacinthe, QC J2S 2M2 Canada; 5 Department of Biological Sciences, Florida International University, 3000 NE 151 St., FL 33181 North Miami, USA; 6 UMR 9175 MARBEC, IRD-CNRS-IFREMER-UM, Université de Montpellier, 34095 Montpellier, France; 7 Department of Anthropology and department of Ichthyology and Fisheries Sciences, Rhodes University, PO Box 94, Grahamstown, South Africa

Correction to: *Scientific Reports* 10.1038/s41598-018-21553-0, published online 27 February 2018

The original version of this Article contained errors.

In the Abstract,

“Since 1988, 86% of shark bite events on ocean-users involved surfers off the leeward coast, where 96% of surfing activities took place.”

now reads:

“Since 1988, 86% of shark bite events on surfers involved ocean-users off the leeward coast, where 96% of surfing activities took place.”

In the Results,

“Of the 43 shark bite events, 67% involved surfers (n = 29), with the remaining 43% involving swimmers (n = 5), spear fishers (n = 5), windsurfers (n = 2), a gillnet fisher (n = 1), and a kayaker (n = 1; Fig. 2a).”

now reads:

“Of the 43 shark bite events, 67% involved surfers (n = 29), with the remaining 33% involving swimmers (n = 5), spear fishers (n = 5), windsurfers (n = 2), a gillnet fisher (n = 1), and a kayaker (n = 1; Fig. 2a).”

“However, this proportion reaches 69% for surfers (Fig. 2b).”

now reads:

“However, this proportion reaches 64% for non-surfers (Fig. 2b).”

Finally, in the Discussion,

“The CFR of 46% for all users and 35% for surfers is very high compared to other countries, such as Australia”

now reads:

“The CFR of 42% for all users and 31% for surfers is very high compared to other countries, such as Australia”

These errors have now been corrected in the PDF and HTML versions of the Article.

