# Supplementary material for: Environmental and anthropogenic factors affecting the increasing occurrence of shark-human interactions around a fast-developing Indian Ocean island
Source: Sci Rep. 2018 Feb 27;8:3676. doi: 10.1038/s41598-018-21553-0 (PMC5829236; doi:10.1038/s41598-018-21553-0)
Supplement: Supplementary file 1 — Supplementary Information [file 41598_2018_21553_MOESM1_ESM.pdf]

Supplementary Information

Environmental and anthropogenic factors affecting the increasing occurrence of shark-human interactions around a fast-developing Indian Ocean island

Erwann Lagabrielle[1,2], Agathe Allibert[3,4], Jeremy J. Kiszka[5], Nicolas Loiseau[6], James P. Kilfoil[5], Anne Lemahieu[1,7]

Supplementary Figure S1

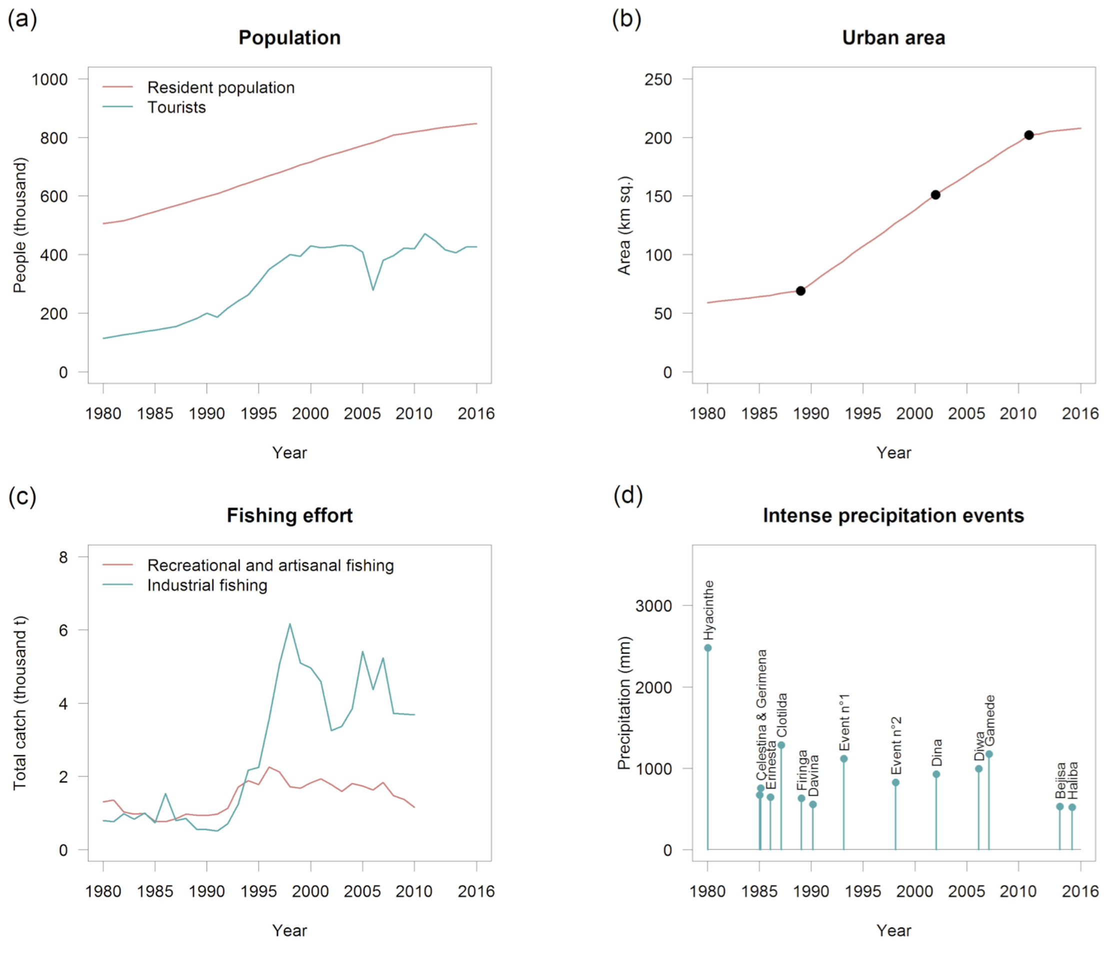

Supplementary Figure 1: From 1980 to 2016, annual number of tourists and resident population in La Réunion (a), urban area (black points indicate measured data) (b), annual reconstructed fish catch in the domestic water of La Réunion (c), intense rainfall precipitation events (>500 mm) (d).

Data sources and methods:

(a) Yearly human demographic data was obtained from the national institute of statistical and economic studies (INSEE) on their website (https://www.insee.fr/). Tourism statistics (number of tourists per year) was obtained from the Regional Tourism Board of La Réunion on their website (www.reunion.fr).

(b) Urban areas in La Réunion were mapped using object-oriented image analysis techniques applied on satellite SPOT 1-4 satellite images degraded to 20 meters resolution for the year 1989 and 2002[32], completed with a SPOT 5 image of the year 2011. Satellite images were collected during the Kalideos project (French National Center of Spatial Studies) providing access to satellite images archives for research purpose. Yearly urban areas data before 1989 and after 2011 were linearly interpolated using population data over the 1980-89 and 2011-2016 periods and the population-urban area ratio of the years 1989 and 2011.

(c) Annual reconstructed fish catch in the domestic water of La Réunion was collected from the Sea Around Us project[33].

(d) Intense rainfall events: intense rainfall events (>500 mm) records were collected from the MeteoFrance website (www.meteofrance.re).

Supplementary Figure S2

(a)

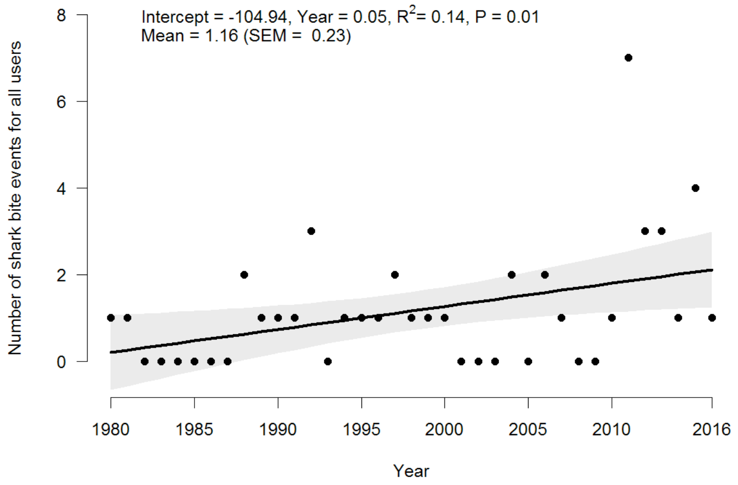

(b)

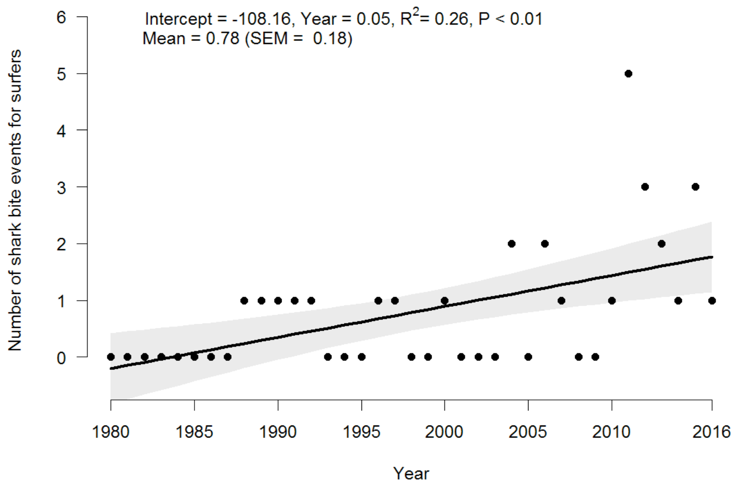

Supplementary Figure 2: Annual shark bite incidence for all categories of ocean users (a) and for surfers only (b) from 1980 to 2016 in La Réunion. Linear regression trendline (dotted grey line) with 95% prediction interval (grey area).

Supplementary Figure S3

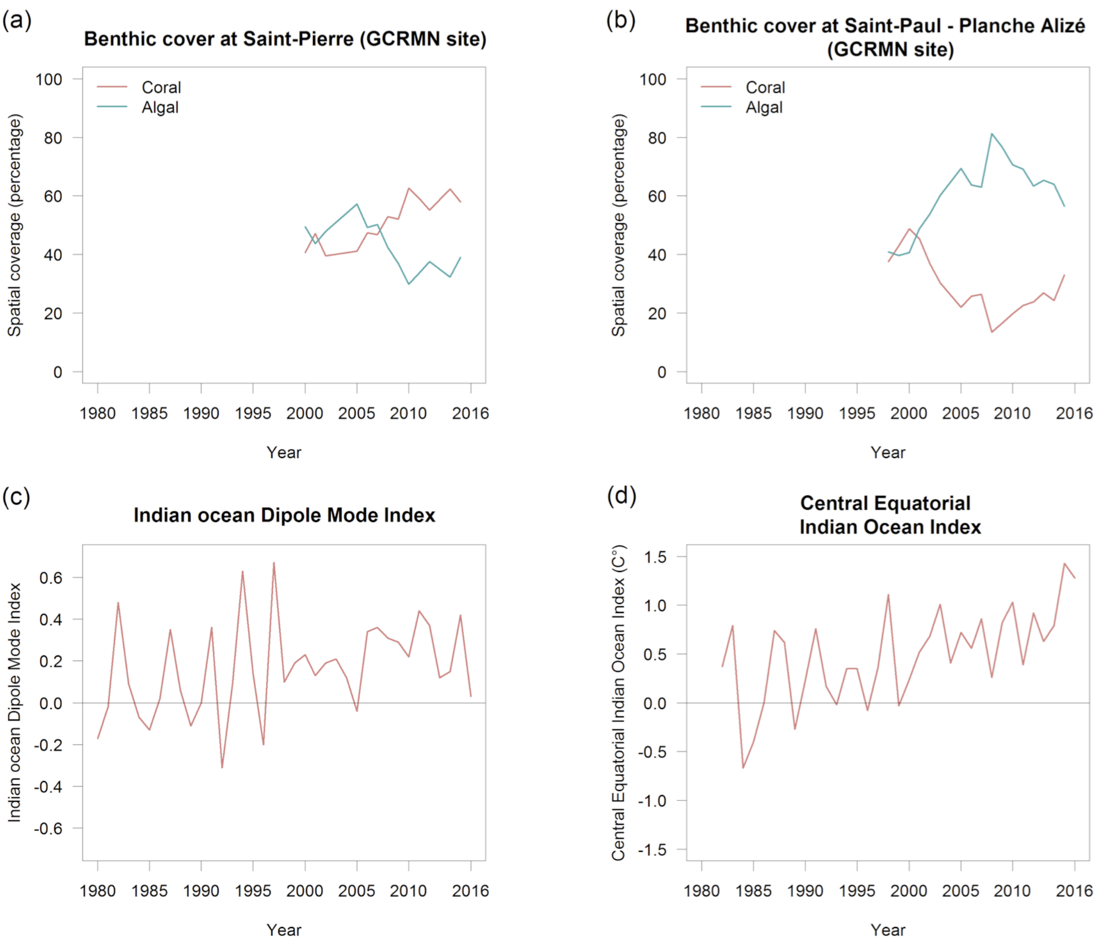

Supplementary Figure 3: Yearly monitoring of benthic substrate spatial cover (coral and algal) at Saint-Paul - Planche Alizé (a) and at Saint-Pierre (b), Indian ocean Dipole Mode Index (DMI) (c), and Central Equatorial Indian Ocean Index (d).

Data sources and methods:

(a) (b) Data from the Global Coral Reef Monitoring Network (GCRMN) method applied in La Réunion[43].

(c) The Dipole Mode Index (DMI) data was retrieved from the Japan Agency for Marine-Earth Science and Technology (JAMSTEC) website (http://www.jamstec.go.jp/). The DMI is an indicator of the east-west temperature gradient across the tropical Indian Ocean, linked to the Indian Ocean Dipole (IOD)[63,63]. Positive IOD is normally characterized by anomalous cooling of SST in the south eastern equatorial Indian Ocean and anomalous warming of SST in the western equatorial Indian Ocean.

(d) The monthly Central Equatorial Indian Ocean Index (CEI) data were retrieved from the NOAA website (http://www.esrl.noaa.gov/psd/). The CEI is a monthly time series of the three-month running average of the standardized monthly mean sea surface temperature anomalies averaged over the region 0° to 15°S latitude and 50°E to 80°E longitude[65]. The climatological base period for the sea surface temperature anomalies is 1971-2000, and the anomalies are standardized with respect to the period December 1981 to present.

Supplementary Figure S4

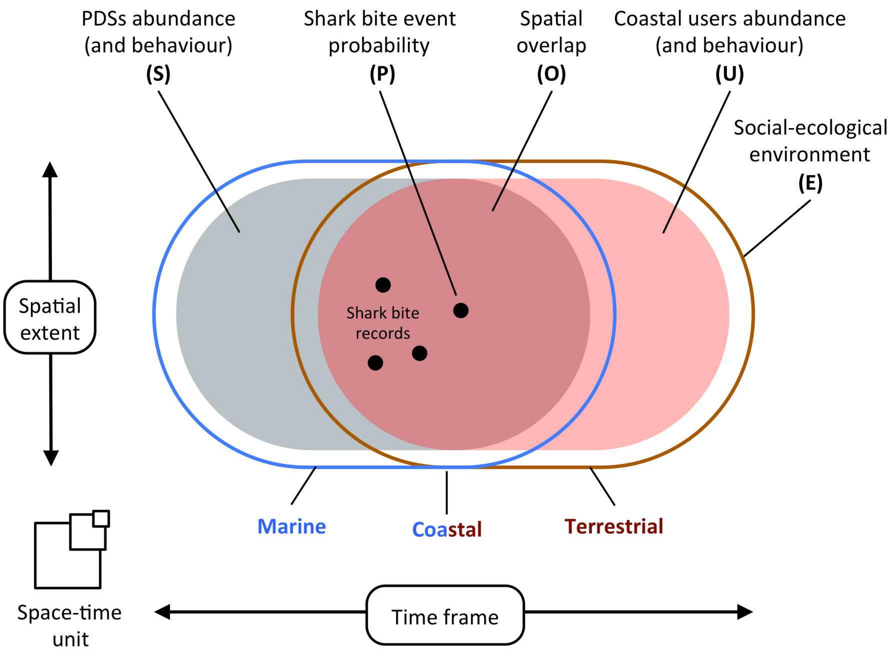

Supplementary Figure 4: Statistical framework used to examine the potential influence of environmental conditions on the occurrence of shark-human interactions. PDS stands for Potentially Dangerous Sharks[19]. The probability of a shark bite on human (P), within a given spatial and time frame depends on the abundance of PDSs (S), the abundance of ocean-users (U), and the overlap (O) between oceans users and PDSs. We also hypothesize that (S), (U) and (O) are influenced by the environment (E). Since shark bites are rare events, the number of bite per unit of space and time can be a function of covariates reflecting S, U, O and E[8].

Supplementary Figure S5

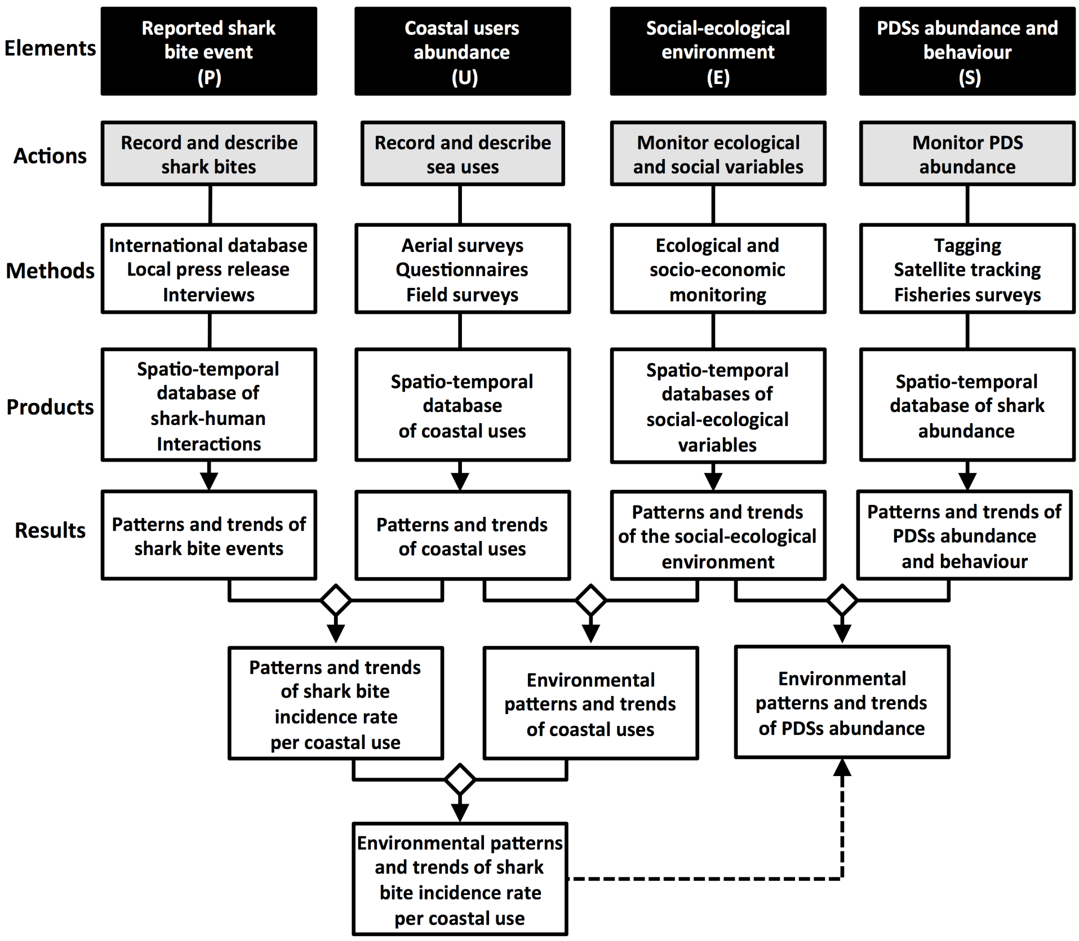

Supplementary Figure 5: Multi-disciplinary framework linking the study of shark bite records to ocean users abundance surveys, shark abundance surveys and social-ecological monitoring.

Supplementary Figure S6

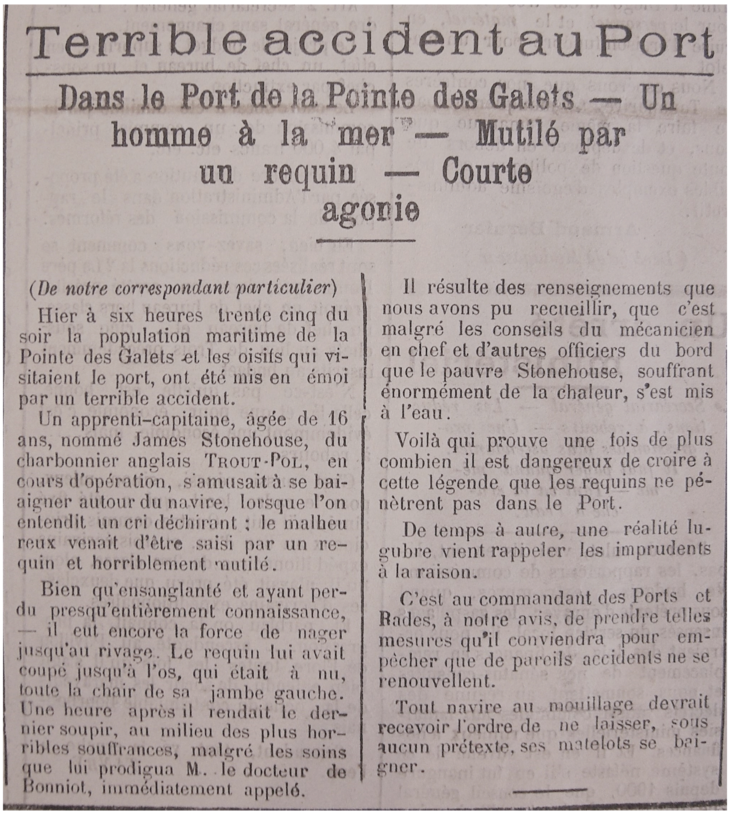

Terrible accident au Port

Dans le Port de la Pointe des Galets — Un homme à la mer — Mutilé par un requin — Courte agonie

(De notre correspondant particulier)

Hier à six heures trente cinq du soir la population maritime de la Pointe des Galets et les oisifs qui visitaient le port, ont été mis en émoi par un terrible accident.

Un apprenti-capitaine, âgée de 16 ans, nommé James Stonehouse, du charbonnier anglais TROUT-POL, en cours d'opération, s'amusait à se baigner autour du navire, lorsque l'on entendit un cri déchirant ; le malheureux venait d'être saisi par un requin et horriblement mutilé.

Bien qu'ensanglanté et ayant perdu presqu'entièrement connaissance, — il eut encore la force de nager jusqu'au rivage. Le requin lui avait coupé jusqu'à l'os, qui était à nu, toute la chair de sa jambe gauche. Une heure après il rendait le dernier soupir, au milieu des plus horribles souffrances, malgré les soins que lui prodigua M. le docteur de Bonniot, immédiatement appelé.

Il résulte des renseignements que nous avons pu recueillir, que c'est malgré les conseils du mécanicien en chef et d'autres officiers du bord que le pauvre Stonehouse, souffrant énormément de la chaleur, s'est mis à l'eau.

Voilà qui prouve une fois de plus combien il est dangereux de croire à cette légende que les requins ne pénètrent pas dans le Port.

De temps à autre, une réalité lugubre vient rappeler les imprudents à la raison.

C'est au commandant des Ports et Rades, à notre avis, de prendre telles mesures qu'il conviendra pour empêcher que de pareils accidents ne se renouvellent.

Tout navire au mouillage devrait recevoir l'ordre de ne laisser, sous aucun prétexte, ses matelots se baigner.

Translation (French to English) :

Terrible accident at Le Port - A man at sea - Mutilated by a shark - Short agony
Yesterday at six thirty-five in the evening the maritime population of Pointe des Galets and the idlers who visited the harbor were thrown into confusion by a terrible accident. // A 16-year-old apprentice-captain named James Stonehouse, of the English coal-minter Trout-Pol, in the course of the operation, amused himself by swimming around the ship, when a heart-rending cry was heard: He was seized by a shark and horribly mutilated. // Though swollen and unconscious, he still had the strength to swim to the shore. The shark had cut him to the bone, which was bare, all the flesh of his left leg. An hour later he breathed his last breath in the midst of the most horrible sufferings, notwithstanding the care lavished on him by the doctor de Bonniot, who was immediately called. // From the information we were able to gather, it was in spite of the advice of the chief engineer and other officers on board that poor Stonehouse, suffering enormously from the heat, got into the water. // This proves once again how dangerous it is to believe this legend that sharks do not penetrate the harbor. // From time to time, a lugubrious reality comes to recall the imprudent to reason. // It is in the opinion of the commander of the harbors and the roadsteads to take such measures as may be necessary to prevent such accidents from recurring. // Any vessel at anchor should be instructed not to allow her sailors to bathe under any circumstances.

Supplementary Figure 6: A shark bite event in La Réunion reported in the daily newspaper *Le Petit Journal de l'île de La Réunion* dated 5th January 1904. This document was retrieved by the Departemental Archives of La Réunion (copyright holder under a CC BY open access license).

Supplementary Figure S7

Year		2010 (48)				2011 (42)				2012 (59)				2013 (15)			
Summer (79), Winter (85)		S (25)		W (23)		S (18)		W (24)		S (28)		W (31)		S (8)		W (7)	
Morning (67), Afternoon (97)		M (13)	A (12)	M (9)	A (14)	M (8)	A (10	M (12)	A (12)	M (12)	A (16)	M (13)	A (18)	M (0)	A (8)	M (0)	A (7)
Holidays period (58)	Week (43)	4	3	4	4	4	3	2	2	3	4	4	3	0	1	0	2
	Week-end (17)	1	1	0	1	0	1	2	2	1	2	1	1	0	2	0	2
Work period (96)	Week (53)	4	4	2	5	2	4	4	4	4	5	4	7	0	3	0	1
	Week-end (51)	4	4	3	4	2	2	4	4	4	5	4	7	0	2	0	2

Supplementary Figure 7: Temporal distribution of flights conducted during the aircraft-based survey of sea-users abundance on the west coast of La Réunion from January 2010 to August 2013 between Etang-Salé in the south and Boucan-Canot in the north (total of 164 flights)[28]. Numbers indicate the count of flights per temporal period. Morning (M) flights were conducted at 10:30. Afternoon (A) flights were conducted at 15:30. Summer (S) is from November to April and winter (W) from May to October.

Supplementary Figure S8

Sector number	2	3	4	5	7	10	15	18	19	21	22	24	26	27	
**Sector name**	**Saint-Paul: Cap Champagne**	**Saint-Paul: Boucan Canot**	**Saint-Paul: Aigrette - Cap Homard**	**Saint-Paul: Grand Fond - Roches Noires**	**Saint-Paul: Les Brisants**	**Saint-Paul: Passe Hermitage**	**Saint-Paul: Trois-Bassins**	**Saint-Leu: Colimacons - Tortue**	**Saint-Leu: Centre - La Gauche**	**Saint-Leu: Sud - Cimetiere**	**Saint-Leu: Pointe au Sel**	**Saint-Leu: Ravine du Trou**	**Etang-Sale: Nord `- Virage**	**Etang-Sale: Bassin Pirogue - Le Brisant**	**Total**
**Year**															
Mean 2010 (*)	0.2 (0.8)	11.3 (12.3)	7 (7.8)	22 (14.9)	0.9 (2.5)	1.6 (3.4)	33.1 (27.4)	7.4 (8.8)	7.8 (12)	2.9 (3.9)	0.3 (1.4)	0 (0)	28.6 (19.6)	7 (8.4)	**130.5 (67.1)**
Mean 2011	0 (0.2)	10.8 (10.5)	5.7 (6.3)	13.8 (11.7)	0.2 (1.4)	3.8 (5.9)	36.9 (23.4)	6.9 (7.2)	12.4 (12.5)	1.9 (3.6)	0.6 (1.8)	0 (0)	36.9 (24.3)	6.7 (7.2)	**136.6 (62.6)**
Mean 2012	0 (0)	0.5 (1.3)	0.4 (1.3)	0.8 (1.7)	0.9 (2.3)	0.8 (2.3)	24.2 (23.9)	4.5 (5.4)	6.9 (10.4)	1.6 (2.8)	0.2 (1.1)	0.1 (0.8)	32.1 (21.7)	9.1 (8.6)	**82.1 (52.5)**
Mean 2013 (**)	0 (0)	0.4 (1.3)	0.6 (2.9)	0.4 (2)	0 (0)	0 (0)	5.2 (9.4)	0.1 (0.4)	0.4 (1)	0.9 (3)	0 (0)	0.3 (1.2)	18.7 (12.4)	1.8 (2.7)	**28.9 (23.8)**
**Year and season**															
Mean Summer 2010 (*)	0.3 (1)	11.8 (13.7)	7.4 (8.6)	21.8 (16)	1.1 (3)	2.5 (4.4)	36.3 (33.9)	9.5 (10.2)	9.3 (12.9)	2.7 (4)	0 (0)	0 (0)	27.5 (20.8)	6.6 (8.6)	**137.8 (77.8)**
Mean Winter 2010	0.1 (0.4)	10.7 (10.7)	6.4 (7.1)	22.3 (13.9)	0.7 (1.7)	0.7 (1.4)	29.6 (17.9)	5.2 (6.3)	6.1 (11)	3 (3.9)	0.5 (2)	0 (0)	29.9 (18.7)	7.5 (8.4)	**122.7 (53.9)**
Mean Summer 2011	0 (0)	14.7 (11.8)	5 (5.2)	17.2 (11.7)	0 (0)	3.7 (6.1)	42.8 (25.1)	7.4 (7.1)	7.9 (9.8)	1.4 (2.1)	0.8 (2.3)	0 (0)	41.4 (30.4)	3.4 (5.9)	**145.8 (65)**
Mean Winter 2011	0 (0.2)	7.9 (8.4)	6.3 (7.1)	11.3 (11.3)	0.4 (1.8)	3.9 (5.9)	32.5 (21.5)	6.5 (7.4)	15.8 (13.4)	2.2 (4.4)	0.5 (1.4)	0 (0)	33.5 (18.6)	9.1 (7.2)	**129.8 (61.2)**
Mean Summer 2012	0 (0)	0.3 (1.1)	0.5 (1.6)	0.5 (1.1)	0.8 (2.4)	1.4 (2.9)	25.5 (22.3)	4 (4.9)	5.1 (10.1)	1.1 (2.5)	0.5 (1.6)	0.2 (1.1)	34.1 (22.5)	10.6 (7.5)	**84.6 (45.6)**
Mean Winter 2012	0 (0)	0.7 (1.5)	0.3 (0.9)	1.1 (2)	1.1 (2.3)	0.4 (1.4)	23 (25.5)	4.8 (5.8)	8.5 (10.5)	2 (3)	0 (0)	0 (0)	30.4 (21.2)	7.7 (9.4)	**79.8 (58.8)**
Mean Summer 2013 (**)	0 (0)	0.8 (1.7)	1.1 (4)	0.8 (2.7)	0 (0)	0 (0)	9.2 (11.5)	0.2 (0.6)	0.7 (1.3)	1.2 (3.9)	0 (0)	0.6 (1.6)	22.2 (12.6)	3.2 (3)	**40.1 (26.2)**
Mean Winter 2013 (**)	0 (0)	0 (0)	0 (0)	0 (0)	0 (0)	0 (0)	0.6 (1.1)	0 (0)	0 (0)	0.6 (1.4)	0 (0)	0 (0)	14.7 (11.4)	0.2 (0.5)	**16.1 (11.6)**
**Year and Day period**															
Mean Morning 2010 (*)	0 (0)	7 (11.4)	4.4 (7.4)	22 (18.1)	0.2 (0.6)	1.6 (3.8)	36.8 (32.3)	7.7 (9.7)	8.4 (15)	2.1 (2.9)	0.1 (0.6)	0 (0)	20.4 (14.7)	6.1 (8.5)	**117.9 (80)**
Mean Afternoon 2010	0.3 (1)	14.9 (12)	9.1 (7.7)	22.1 (12)	1.5 (3.2)	1.7 (3.2)	30 (22.6)	7.2 (8.1)	7.2 (9.1)	3.5 (4.6)	0.3 (1.8)	0 (0)	35.6 (20.7)	7.8 (8.4)	**141.2 (53.3)**
Mean Morning 2011	0.1 (0.2)	8.9 (8.9)	5 (5.2)	13.8 (12.1)	0 (0)	3.4 (5.8)	39.1 (25.1)	5 (6.5)	12.6 (13.2)	0.9 (1.5)	0.5 (1.7)	0 (0)	26.9 (21.6)	5.5 (6.1)	**121.5 (61.2)**
Mean Afternoon 2011	0 (0)	12.6 (11.7)	6.4 (7.2)	13.9 (11.7)	0.4 (1.9)	4.2 (6.1)	35 (22.1)	8.6 (7.5)	12.2 (12.1)	2.7 (4.6)	0.7 (1.9)	0 (0)	46 (23.5)	7.7 (8.1)	**150.4 (62)**
Mean Morning 2012	0 (0)	0.7 (1.5)	0.7 (1.8)	1.3 (1.9)	0.8 (1.7)	1.1 (2.7)	35 (25.8)	5.2 (6.2)	7.2 (11.2)	2.2 (3.4)	0.1 (0.4)	0.2 (1.2)	31.2 (22.4)	10.2 (9.5)	**96 (56.5)**
Mean Afternoon 2012	0 (0)	0.4 (1.1)	0.1 (0.6)	0.5 (1.4)	1.1 (2.7)	0.6 (1.8)	16.2 (19.1)	3.9 (4.7)	6.6 (9.9)	1.1 (2.2)	0.3 (1.5)	0 (0)	32.8 (21.5)	8.2 (7.9)	**71.9 (47.7)**
Mean Morning 2013 (**)	0 (0)	0.4 (1)	1.1 (4.1)	0.7 (2.8)	0 (0)	0 (0)	7.5 (12.2)	0.1 (0.3)	0.4 (1.1)	1.3 (4.1)	0 (0)	0 (0)	18.3 (12.4)	1.9 (2.8)	**31.6 (28)**
Mean Afternoon 2013	0 (0)	0.5 (1.6)	0.1 (0.5)	0.1 (0.5)	0 (0)	0 (0)	2.9 (4.7)	0.1 (0.5)	0.3 (0.9)	0.5 (1.2)	0 (0)	0.7 (1.6)	19.1 (12.9)	1.7 (2.6)	**26.2 (19.3)**

(*) Morning flights in summer 2010 were conducted at 8:30. A corrective coefficient was applied to those values based on the mean morning/afternoon ratio of summer 2011.

(**) No morning flights were scheduled in 2013. Morning values for the year 2013 were inferred based on afternoon values for the year 2013 and morning/afternoon ratio of the year 2012 (per season).

Supplementary Figure 8: Mean instantaneous count of surfers (mean ± SEM) measured througout the aircraft-based survey of sea-users conducted on the west coast of La Réunion from January 2010 to August 2013 between Etang-Salé and Boucan-Canot (total of 164 flights)[28]. Morning flights were conducted at 10:30. Afternoon flights were conducted at 15:30. Summer is from November to April and winter from May to October.

Supplementary Figure S9

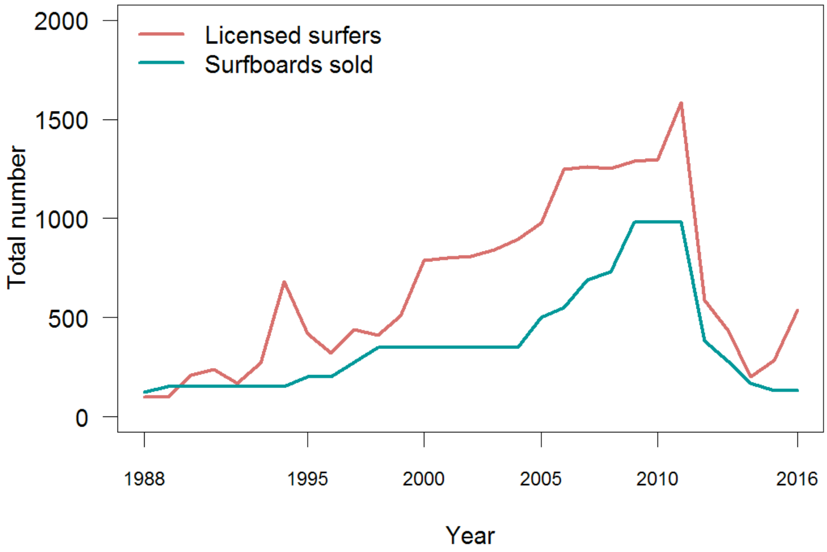

Supplementary Figure 9: Number of licensed surfers registered at the Surf League of La Réunion and number of surfboard sold in La Réunion from 1988 to 2016.

Data sources and methods:

Surf licensees are registered at the Surf League of La Réunion (created in 1984). Surf licensees registered through profit surf schools are excluded from summary licensees statistics since 2007. The annual number of surf licensees increased sporadically under the following circumstances:

- 1994 : The Surf League of La Réunion and the Association of Surfing Professionals organized the "Réunion Surf Pro", an international surfing competition event.
- 2012: The Surf League of La Réunion organized a "licensing" communication campaign to increase the number of licensees with the objective of influencing the negotiation of shark risk mitigation measures.
- 2015-16: A license is mandatory for surfers surfing in experimental surf zone "zonex" under the "vigie requin renforcée (VRR)" programme.

The number of surfboards sold in La Réunion was estimated based on data collected from the main surfshop owners in La Réunion (period of activity into parenthesis): Mickey Rat (1987-present), Choka (1997-2010), Delbourg (1989-2012), Iluka (2005-2011), Karv (2007-present), Inside (2005-2015), Esprit Surf surfshop (2009-2011) and Billabong surfshops (1998-2017).

Supplementary Figure S10

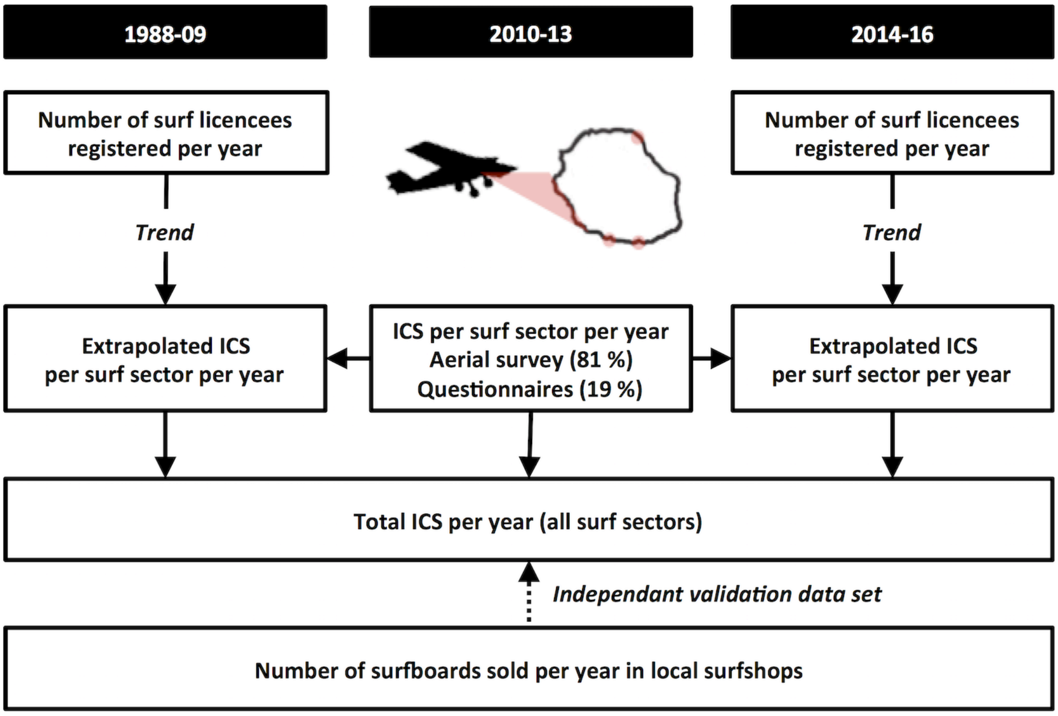

Supplementary Figure 10: Overview of data and methods combined to assess, extrapolate and validate the instantaneous count of surfers (ICS) in La Réunion from 1988 to 2016.
